# The risk of biological race

**DOI:** 10.1007/s40656-025-00684-4

**Published:** 2025-07-30

**Authors:** Celso Neto

**Affiliations:** 1 Egenis - Centre for the Study of the Life Sciences, University of Exeter, Byrne House, St. Germain Road, Exeter, EX4 4PJ UK; 2 Department of Social and Political Sciences, Philosophy and Anthropology, University of Exeter, Exeter, UK

**Keywords:** Race, Biological race realism, Epistemic risk, Ethical risk, Non-epistemic values

## Abstract

Biological race realism (hereafter BRR) is the view that humans form biologically distinct groups. In recent years, Quayshawn Spencer has offered one of the most elaborate versions of that view, but his theory faces several problems (Spencer in Philos Stud 159:181–204, 2012; Spencer in J Philos 111:1–23, 2014; Spencer in 52:46–55, 2015; Spencer in Glasgow, Haslanger, Jeffers, Spencer (eds) What is race? Four philosophical views, Oxford University Press, Oxford, 2019a; Hochman in J Philos 110:331–351, 2013; Glasgow et al. in Glasgow, Haslanger, Jeffers, Spencer (eds) What is race? Four philosophical views, Oxford University Press, Oxford, 2019; Jackson in Philos Theory Pract Biol 14, 2022. https://doi.org/10.3998/ptpbio.2630; Winsberg in Biol Philos 37:46, 2022; Msimang in Philos Pap 51:115–145, 2022; Kalewold in Metaphysics of race, Cambridge University Press, Cambridge, 2024; Berenstain forthcoming). In this paper, I raise another problem for Spencer’s BRR, arguing that his theory does not fully consider how social, political, and moral values influence the metaphysics of race. Spencer’s BRR involves significant epistemic and ethical risks, and these risks indirectly impact Spencer’s metaphysical conclusions. I rely on the “science and values” literature to show this and engage with STS and anthropology literature (Douglas in Inductive risk and values in science, 2000. http://www.journals.uchicago.edu/t-and-c; Douglas in Science, policy, and the value-free ideal, Pittsburgh University Press, Pittsburgh, 2009; Brown in Philos Sci 80:829–839, 2013; Brown in Current controversies in values and science, Routledge, Milton Park, 2017; Biddle and Kukla Explor Induc Risk Case Stud Values Sci 1:215–238, 2017; Elliot and Richards in Exploring inductive risk: case studies of values in science, Oxford University Press, Oxford, 2017). This analysis raises broader questions about the relationship between values, social responsibility, and metaphysics. Previous criticisms of Spencer’s BRR have barely touched on those questions. Hence, by critically discussing problems with Spencer’s already troubled view, my main goal is to open the debate for such important questions.

## Introduction

Biological race realism (hereafter BRR) is the view that humans form biological races. This view has a long history related to racism and colonialism (Bernasconi & Lott, 2000; James & Burgos, 2020). For instance, the so-called “racialist conception of race” was prominent in Europe and North America during the 19th Century. This version of BRR states that human races form a hierarchy according to their distinct capacities (Hardimon, 2017; Taylor, 2013). Each racial group would be defined by immutable biological essences shaping individuals’ physical, behavioral, intellectual, and moral traits. In this sense, racial membership would be highly explanatory. It would explain why and how humans differ in intelligence, empathy, loyalty, and many other meaningful traits. These differences would determine the superiority of some races over others.

Science has debunked the racialist conception of race, but other versions of BRR have surfaced recently (Andreasen, 2004, 2005; Kitcher, 1999; Pigliucci & Kaplan, 2003; Hardimon, 2003, 2017; Spencer, 2014, 2019a; Glasgow & Woodward, 2015). Many of these versions reject ideas of racial hierarchy, superiority, and essences. They also deny that racial groups necessarily differ in meaningful traits. *Minimalist* (or deflationary*)* versions of BRR have gained special attention in the last decade (Hardimon, 2003, 2017; Spencer, 2012, 2014, 2015, 2018b, 2019a). Minimalism proposes that racial groups differ mainly in superficial phenotypic attributes, such as skin color and hair texture. Thus, racial membership is not explanatory in the way proposed by the racialist conception of race. At the same time, minimalism defends that medicine and other areas of biology still benefit from talking about race and particular racial groups. For example, the proponents of minimalist argue that identifying a patient as “White” or “Black” can be useful when diagnosing Cystic Fibrosis, a condition to which white populations are more susceptible than other groups (Hardimon, 2017, p. 156; see also Spencer, 2018a).^1^ The apparently important uses of racial language and classifications in biology is often presented as a motivation to examine whether ‘race’ denotes a real biological kind. Frequently, BRR proponents move from the epistemic utility of those classifications to kind realism (Hardimon, 2017; Spencer, 2019a). These kinds would correspond to a significant and non-arbitrary type of entity or subdivision in nature.^2^ Different racial groups would be instances of that kind. Moreover, given the relevance of talking about specific racial groups (e.g., “White” and “Black”), these groups are also treated as biological kinds themselves (Spencer, 2019a).

Arguments against minimalist versions of BRR appear in the philosophical literature (Glasgow, 2009; Hochman, 2013; Spencer, 2018a; Glasgow et al., 2019; Jackson, 2022; Winsberg, 2022; Kalewold, 2024, Berenstain forthcoming). One class of arguments focuses on the idea of real biological kinds (or entities) (Gannett, 2010; Hochman, 2013; Kalewold, 2024; Maglo, 2011). Gannett (2010) argues that real biological kinds must serve various epistemic goals in biology, but the kind ‘race’ does not. She and other scholars challenge the epistemic utility of using race and racial groups in biological science, particularly medicine (Root, 2003; Yudell et al., 2016). According to these arguments, race is not a real biological kind because the practice of grouping humans into races is not useful *enough* or *in the right way*. Minimalists push back on these arguments by specifying what is meant by “epistemic utility” and defending that biological kinds can be real even if they are only modestly or marginally useful (Hardimon, 2017; Spencer, 2014, 2019a). This strategy is carefully developed by Quayshawn Spencer, who grounds his minimalist BRR on a new theory of biological kinds (2012, 2014, 2018a, 2019a).

In this paper, I examine Spencer’s version of BRR to explore a problem missed by previous criticisms. While several scholars focus on the issue of epistemic utility, I consider the role of non-epistemic considerations in minimalist BRR. More specifically, I argue that Spencer relies on epistemic utility at the expense of non-epistemic considerations and, thus, fails to fully recognize that social, political, and moral values influence the metaphysics of race.^3^ Moreover, I argue that these values should influence this area even when philosophers are supposedly engaged in more descriptive (in contrast to ameliorative, conceptual engineering) projects in the metaphysics of race. Kitcher (2007) raised a similar point in the past, arguing that the legitimacy of race depends on the epistemic and non-epistemic utility. Spencer (2012) rejected Kitcher’s approach as relying on controversial assumptions about the fact-value distinction.

The argument presented in this paper is not as easy to dismiss. While social, political, and moral considerations might not justify the reality of races (or lack thereof), they can indirectly influence the metaphysical reasoning around those justifications. This paper is the first effort in identifying and analyzing this influence.^4^ My aim is not simply to criticize Spencer’s view, as this has been done rather convincingly (Hochman, 2014; Kalewold, 2024; Winsberg, 2022). Instead, I explore Spencer’s flawed theory as a *case study* that can help us think about the relationship between the metaphysics of race and non-epistemic considerations. Here I rely on the “science and values” literature and the notions of inductive, epistemic, and ethical risk (Douglas, 2000, 2009; Brown, 2017; Biddle & Kukla, 2017; Elliot & Richards, 2017). Once one realizes the complex relationship between these types of risks and Spencer’s theory, one can recognize how non-epistemic factors influence his metaphysical conclusions. This recognition raises broader questions about values, responsibility, and metaphysics of race. After all, given that metaphysical reasoning about race involves non-epistemic influence, responsible metaphysics about race should be sensitive to them.

In the next section, I present Spencer’s BRR (Sect. 2). I offer a close and charitable presentation of Spencer’s reasoning while acknowledging its numerous problems. Then, I discuss how Spencer deals with a few objections to his view (Sect. 3). These objections focus on epistemic utility and how it relates to non-epistemic value judgments. Thereafter, I introduce the notions of inductive, epistemic, and ethical risk present in the “values and science” literature (Sect. 4). These notions enable me to propose that a socially responsible metaphysics of race should consider those risks. Literature from anthropology and STS indicate that such risks are indeed very real. Given such risks, I show that metaphysicians of race like Spencer have an unfinished task. At the very least, they must provide an extremely clear and convincing case for the epistemic utility of race that outweighs the risks. So far, the supposed epistemic utility for BRR is not that clear or convincing. In conclusion, I invite philosophers to explore the intersection between metaphysics and risk, considering what it means to do metaphysics of race in a socially responsible way.

## Spencer’s biological race realism and its problems

Two preliminary qualifications are necessary to understand Spencer’s minimalist version of BRR. First, it concerns the reality of race and racial groups if by “race” and “racial groups” we mean the types of phenomena usually identified by people as such in their daily lives. In other words, the question at stake is whether people talk about something real when they use racial terms—such as “race”, but also “Black” and “White”—in ordinary contexts (Spencer, 2018a, 2018b). Spencer’s answer to the question is a qualified “yes.” Second, this answer concerns the reality of race as ordinarily understood in the United States. Spencer has nothing to say about race in Brazil, South Africa, or anywhere else. Thus, the scope of his view is quite circumscribed to begin with: it concerns what people in the US talk about when they use racial terms in everyday contexts.

The Census provides paradigmatic examples of how people in the US talk about race. The Office of Management and Budget (OMB) is responsible for organizing the Census and thus determining/defining racial terms (Spencer, 2019a, 2019b). These terms are “White,” “Black” or “African-American,” “Asian,” “American-Indian,” or “Alaska Native,” and “Native Hawaiian” or “Pacific Islander.” This terminology is widespread in various social contexts in the United States. For instance, it figures in healthcare surveys, medical records, college and job applications, and housing and aid program questionnaires (Spencer, 2019a, p. 79). Hospitals, companies, universities, and many other social institutions adopt OMB’s racial terms and definitions. Hence, on many occasions, people follow the OMB conventions whether they recognize them or not. For this reason, Spencer argues that when people use a racial term like “Black” in the United States, they are frequently talking about the same group of people that would count as Black/African-American in the OMB Census (2019a, pp. 82–83).

Spencer argues that the racial terms in the OMB Census frequently refer to what is known in population genetics as the five main “human continental populations” or “geographical populations” (Spencer, 2019a, p. 99). These populations have distinctive geographical origins at the sub-continental level. They are: Black Africans with origins in Sub-Saharan Africa, Eurasians with origins in Eurasia (West Europe, Middle East, and South/Central Asia), Asians with origins in East Asia, Native Americans (or Amerindians) with origins in Alaska and North America, and Oceanians with origin in Oceania. This division maps into the racial terminology and classification proposed by the OMB, as described above. The terms “Black” and “African-American” refer to the Black African origin population; the term “Asian” refers to the East Asian origin population; the term “White” refers to the Eurasian origin population; the terms “American Indian” and “Alaska Native” refer to the Native American origin population; and the terms “Hawaiian Native” and “Pacific Islander” refer to the Oceanian origin population.

So far, I have simply described Spencer’s views on how racial terms are used in US everyday contexts. However, these views face significant challenges. Spencer argues that people use racial terms in ways that conform to the OMB and that such terms pick out continental populations. It is hard to defend these points without extensive empirical evidence from linguistics, but Spencer does not provide much of it (Jackson, 2022). Moreover, while sometimes people might follow OMB conventions, they often and actively resist them. Recent changes to the OMB racial terminology (e.g., the inclusion of the MENA racial category) result from people’s dissatisfaction with OMB (Haslanger, 2019). Furthermore, many scholars reject the idea that racial terms in the US ordinarily refer to the human continental populations as described by geneticists (Glasgow, 2009; Jeffers, 2019; Mallon, 2006). This is the famous *mismatch objection*. While human continental populations might be “real” in some sense, they do not correspond to the racial groups that people in the US are referring to by racial terms in everyday situations. Spencer tries to deal with this objection, but counterexamples surmount (e.g., Jeffers, 2019; Spencer, 2019a). Finally, Spencer’s characterization of racial discourse relies on a referentialist theory of OMB racial terms, but this theory faces numerous counterexamples (Jackson, 2022; Winsberg, 2022). If these terms pick out human continental populations, this would be a massive coincidence in how ordinary people and geneticists talk. The upshot is that Spencer’s BRR rests on unsubstantiated assumptions about language.

For the sake of argument, let’s assume that racial terms in US ordinary contexts can sometimes refer to the five human continental populations. This assumption enables Spencer to interpret the question of race realism as a question of human continental population realism.^5^ Thus, the question now becomes whether the category ‘human continental population’ refers to a real biological kind and whether any specific human continental population (e.g., Eurasian, Asian) corresponds to a real biological kind as well. Below, I describe how Spencer offers a positive answer to these questions. This requires understanding how he interprets some studies in human genetics. Then, I will consider the problems with these studies, Spencer’s interpretation, and his argument more generally.

Spencer turns to work on the genetic structure of human populations to answer that question (Rosenberg et al., 2002, 2005; Pemberton et al., 2013). In their 2002’ landmark study, Noah Rosenberg and colleagues used 377 autosomal microsatellite markers from 1,056 individuals in 52 populations across the globe. In 2005, they expanded this data and used 783 microsatellites and included 210 insertion/deletion polymorphisms (another type of genetic marker). In 2013, scientists analyzed microsatellites of 5795 individuals from 267 worldwide populations. In all these studies, Rosenberg and colleagues relied on a specific algorithm (STRUCTURE) to cluster individuals into groups based on genetic similarity. In this algorithm, scientists decide how many groups the genetic similarity analysis should generate. The result of these particular studies was the following: when scientists decide to divide humans into five groups of genetic similarity, these groups seem to roughly match the five human continental populations—Black Africans, Eurasians, East Asians, Native Americans, and Oceanians (Spencer, 2019a, pp. 96–99). This “match” is imperfect, however, to say the least. For example, Central and South Asians end up included in the Eurasian category, but it is unclear why they should be grouped with Europeans as one “human continental population.” Yet, Rosenberg and colleagues consider the result meaningful. For instance, depending on the available genetic data set and sampling decisions, they argue that one can use STRUCTURE to produce reliable predictions about the *self-declared* continental ancestry origin of individuals (Feldman & Lewontin 2008; Hardimon, 2017).^6^ Moreover, scientists use STRUCTURE primarily as an exploratory tool to generate hypotheses about population structure (Pritchard et al., 2000; Griesemer & Barragan, 2022). The algorithm is useful to generate working hypotheses about differences in allele frequencies across human continental populations (and intra-continental sub-populations).

At this point, it is important to be critical about science and how Spencer uses it. Spencer relies exclusively on the work by Rosenberg and collaborators, but other population geneticists and scientists raise several issues concerning those studies (Serre & Paabo, 2004; Tishkoff & Kidd, 2004; DeSalle & Tattersall, 2018). The algorithm STRUCTURE is designed to generate clusters and, thus, it will necessarily emphasize genetic differentiation among humans. However, it is well-known that genetic differences among humans are extremely small and increase gradually with geographical distance (Serre & Paabo, 2004; Fujimura et al., 2014). If we systematically sample DNA from individuals across the globe, we will find *clines* rather than clusters. Moreover, if we extensively sample DNA from individuals in Africa, STRUCTURE will produce clusters (as it always does) that look rather different (Tishkoff et al., 2009). The reason is that most genetic diversity exists within Africa. So, instead of five clusters each representing one continental population, many clusters would represent different African groups. One important upshot of these criticisms is that the results obtained by Rosenberg et al., (2002, 2005) are *not* surprising in an important sense: they only obtain given specific decisions of where to sample DNA, what variants to include, etc. (Reardon, 2005).

Additionally, it is important to notice that STRUCTURE requires stipulating the number of clusters beforehand. Hence, there is nothing distinctively natural about grouping humans into five rather than six, seven, or any other number of clusters (Winsberg, 2022). For reasons like this, one should not assume that human continental populations represent a privileged or significant level of genetic differentiation (Hochman, 2014). In other words, while human continental populations have slight genetic differences, they cannot sufficiently capture human genetic variation and might even distract us from offering a fully accurate representation of this variation.^7^ When Spencer decides to focus on K = 5 and Rosenberg et al. (2002), he ends up ignoring key facts about human genetic variation, such as that only an extremely low amount of variation (1.53%) is not explained by geographical distance (Hochman, 2014).

Despite the criticisms above, Spencer argues that the set of five human continental populations is real. He states that both the set as a whole and each individual continental populations are real biological kinds (2019a, p. 100). To justify these claims, Spencer relies on a particular theory of what counts as real biological kinds (Spencer, 2012, 2016, 2019a). According to him, real biological kinds must satisfy three conditions. First, they must be part of a well-ordered scientific research program (hereafter SRP). Second, they must be useful for producing scientific generalizations in this research program. Third, these generalizations must be warranted in that research program such that the categories underwriting the generalizations are epistemically justified in that program. Spencer claims that the set of five human continental populations satisfies these conditions, as I will explain next. After explaining how Spencer arrives at those claims, once again I raise problems to his argument.

A well-ordered SRP is a paradigmatic example of productive and reliable scientific practice. This type of research program has significantly higher chances of long-term success than its potential competitors (Spencer, 2012, p. 192). For instance, a well-ordered SRP has coherent and well-motivated research aims. There is nothing incoherent or contradictory between the aims of the research and its other components, such as methodologies, theories, and experiments. Moreover, a well-ordered SRP has “competitive predictive power” (Spencer, 2016, p. 168). This power involves the capacity of the SRP to predict known and new phenomena with success that is at least on par with rival research programs. Finally, well-ordered SRPs must routinely cross-check their results. The studies within these SRPs must be replicated with slight changes in background assumptions, methodologies, instruments, etc. This practice ensures that the results from those SRPs are robust and reliable.

Spencer argues that continental human populations are part of a well-ordered scientific research program, namely the study of the genetic structure of human populations by population genetics (2019a, p. 95). More specifically, the set of studies conducted by Noah Rosenberg exemplifies that well-ordered SRP (Rosenberg et al., 2002; Pemberton et al., 2013; Rosenberg et al., 2005). According to Spencer, these studies have well-motivated research aims, such as understanding the genetic diversity of human populations, and there is nothing internally incoherent in the work of Rosenberg and colleagues. Spencer also claims that those studies have competitive predictive power by generating predictions and new hypotheses to be tested. One example would be the hypothesis “the subdivision of humans into five genetic clusters corresponds to the five human continental populations”. Note, however, that this hypothesis is true only if we collapse Europeans, South Asians, and Central Asians into a single category (Sect. 2). Finally, the studies conducted by Rosenberg and colleagues receive constant cross-checking (Rosenberg et al., 2002; Pemberton et al., 2013; Rosenberg et al., 2005; Mallick et al., 2016).

The second condition of real kinds is the capacity to produce scientific generalizations in the respective well-ordered SRP. These kinds must be epistemically useful in SRPs by underwriting generalizations. Examples would be the kinds *allele* and *gene* in classic Mendelian genetics (2016). These kinds ground scientific generalizations, such as the so-called Mendel’s law of segregation: “alleles of the same gene segregate into different gametes during gametogenesis” (Spencer, 2016, p. 166). Without acknowledging alleles and genes, it would have been impossible to produce this generalization. Likewise, the set of human continental populations presumably grounds generalizations in population genetics. This set (Black Africans, Eurasians, East Asians, Native Americans, and Oceanians) helps Rosenberg and colleagues to “formulate a theory about human population structure” (2019a, p. 99). This theory states that the division of human continental populations matches the genetic subdivision of humans into five main genetic clusters. In other words, the category of ‘human continental population’ and the reference to particular human continental populations are useful because—at the very least—they enable some geneticists to formulate hypotheses about genetic divisions between humans.

To be real, a kind must also be *adequately epistemically justified* in an SRP (Spencer, 2012, p. 189). This justification depends on the relation between the kind, the underwritten generalizations, and the epistemic values of the well-ordered SRP. According to Spencer, a kind is justified if its underwritten generalizations can predict or explain things according to the epistemic standards of the SRP. For example, Calvin Bridges used the concept of the chromosomal genes in Mendelian genetics to formulate a theory (generalization) about the segregation of sex chromosomes in *Drosophila amphelophila* (Spencer, 2016, p. 167). His theory has proven to be adequate in the context of Mendelian genetics because it satisfies two central epistemic values of this field, namely empirical accuracy (adequacy) and quantitative precision (how similar are the quantitative measurements of the same phenomenon). Hence, Bridge’s theory offers a legitimate explanation of sex-chromosome segregation in that species of *Drosophila*. The consequence is that the category of chromosomal genes is epistemically justified in Mendelian genetics.

Spencer argues that the set of human continental populations is adequately justified in the SRP of population genetics (2019a, p. 97). According to him, Rosenberg and colleagues refer to the set of five continental populations to formulate and test hypotheses about genetic distribution across humans. Spencer does not go into detail here, but one example of such a hypothesis would be “the set of five human continental populations is the population subdivision at K = 5 in humans” (Spencer, 2019a, p. 99). Furthermore, according to Spencer, this hypothesis is empirically accurate and quantitatively precise, as it has been tested repeatedly. While this hypothesis might seem to meet these requirements and other standards of population genetics, Spencer does not offer explicit evidence for his claims. For instance, as I mentioned previously, that hypothesis could only be accurate if one assumes that Europeans, South Asians, and Central Asians as part of the same human continental population (Sect. 2). Spencer offers no other examples, but he claims that similar hypotheses can be generated for each of the particular five human continental populations (Spencer, 2019a, p. 100). What he seems to have in mind here are hypotheses such as “One of the population subdivisions at K = 5 in humans corresponds to the African continental population.” Hence, Spencer concludes that the K = 5 subdivision is real and each one of the five continental populations is a real biological kind.

As I have described above, Spencer relies on a theory of real kinds to conclude that races (i.e., the five human continental populations) are biologically real. However, how strong is his theory and conclusion? Again, it is important to be critical. First, Spencer’s theory is entirely based on the set of studies generated by Noah Rosenberg and colleagues. While these studies are mainstream and useful for specific purposes, I already suggested that population geneticists take different approaches to studying the genetic structure of populations (Tishkoff et al., 2009). The work done by Rosenberg and colleagues is not representative of the whole field of population genetics. Recently, there has been a clear pushback against using human continental populations in that study (Coop, 2022; Lewis et al., 2022). Second, STRUCTURE-based studies typically generate numerous clusters at different levels of granularity (Novembre & Stephens, 2008). This is in part because these are exploratory studies (Pritchard et al., 2000; Griesemer & Barragan, 2022). Scientists are successfully formulating and testing numerous hypotheses and alternative scenarios about the division of humans into genetic clusters. So, if Spencer commits to the reality of the set of five human continental populations, he would also have to commit to the reality of very many other sets of populations (Winsberg, 2022). This promiscuity is not necessarily a problem. Nevertheless, it shows that his metaphysical theory of real kinds is very thin, i.e., the reality of entities is easy to obtain and many entities should be considered “real.” Reality claims lose epistemic and normative force. In other words, one is faced with the question of what is the philosophical gain of making such weak (thin) claims about the reality of races, particularly when one considers the social and ethical risks that these claims entail. I will return to this point in future sections.

For now, it suffices to say that Spencer’s BRR faces numerous challenges. This view relies on a set of genetic studies that (by design) overlook the main facts about human genetic diversity. Spencer combines these studies with flawed linguistic assumptions and thin metaphysics. As a result, his defense of BRR ignores the main facts about human genetic diversity (Hochman, 2014).

In making his argument, it is also worth noticing that Spencer is forced to reject how two professional groups describe their own work. First, Spencer must reject how the OMB characterizes the purposes and definition of racial terms in the Census. OMB understands these terms as referring to social constructs rather than biological races (Jackson, 2022; Winsberg, 2022). Second, Spencer rejects how Rosenberg et al., (2002, 2005) characterize human continental populations and how they relate to race. These scientists do not agree that human continental populations correspond to races (2005). They emphasize that human continental populations rely on a technical notion of genetic ancestry and similarity, but biological races do not. Spencer’s argument rejects this differentiation. He assumes that scientists quite literally do not know what they are talking about.^8^

## Epistemic utility and further problems for biological race realism

In this section, I look closely at a crucial aspect of Spencer’s BRR: its appeal to epistemic utility to defend the reality of race and racial groups (i.e., both the set of five human continental populations and each of its populations). I will survey further problems against Spencer’s theory and how he rejects the influence of non-epistemic factors in his metaphysics of race. This analysis will set the stage for the next section, where I argue that non-epistemic factors do (and should) influence the metaphysics of race.

In the last decades, some scholars have resisted BRR in general by pointing out that talking about race and racial divisions is not very useful in biology (Gannett, 2010; Hochman, 2013; Maglo, 2011). Biologists cannot explain much when appealing to those groupings. Hence, one might conclude that race and races do not deserve to be called “biologically real.”

Spencer is aware of this resistance. He replies to it with a “parity of reasoning” argument. According to him, race and racial groups should be considered real for the same reason that any other entity in science is treated as real: they must be “epistemically useful and justified in a well-ordered research program” (2019a, p. 95). The reality of entities in science cannot depend on them being very useful to scientists because many entities in science are not fundamental or central to scientific theories and predictions. As an example, Spencer cites the case of the 93C allele from the TYRP1 gene and element 17 in chemistry. The 93C allele’s only function is coding for blond hair in some Melanesian people, and thus, it does not help scientists to explain or investigate much (2019a, p. 95). Likewise, element 117 has nuclear instability, which explains why chemists do not do many things with it (2014, p. 1035). Nonetheless, Spencer argues, scientists do not deny that the 93C allele and the element 117 exist. These entities have very modest epistemic utility, but this would be enough to vindicate their reality. The upshot is that, under the risk of logical inconsistency, race and racial groups (i.e., the five human continental populations) can be real if they have modest epistemic utility.

At this point, more problems arise for Spencer’s theory. First, his actual defense of the modest epistemic utility of race and racial groups is brief and under-described. It relies on a single example of how the expression “five human continental populations” might figure in a testable hypothesis (Sect. 2). Second, Spencer’s notion of epistemic utility is far too modest. Kal Kalewold (2024) convincingly explores this issue from several angles. He shows that (i) many arbitrary entities are also minimally explanatory and, thus, epistemically useful; (ii) real kinds typically contribute to scientific understanding by establishing explanatory connections with other such kinds and, thus, helping to explain a wide range of phenomena; (iii) the division of races as five human continental populations can only offer limit explanatory work because the properties of each population are not reasonably portable or projectible; (iv) the phenomena allegedly explained by appeal to the five human continental populations are better explained by other mechanisms or levels of human population subdivision (2024, p. 28). Explanations referring to the five continental populations lack specificity and other well-known features of scientific explanations (2024, p. 29). These are features that might be present in explanations involving the TYRP1 gene, the 93C allele, and other putative biological kinds, but it is not present in the case of race and races *qua* human continental population. The upshot is that Spencer’s appeal to modest epistemic utility is not enough to vindicate BRR. Thus, besides all the problems discussed in the previous section, Spencer’s BRR moves too quickly from epistemic utility to metaphysical conclusions.

Some philosophers of race argue that the reality of race and racial divisions is not only a matter of epistemic utility. Phillip Kitcher (2007) adopts this viewpoint. According to him, race and racial groups are legitimate (“real”) only if reference to them is useful to society rather than specific fields of science. This reference must be more beneficial than harmful across different areas of society. Moreover, these benefits are not merely epistemic. In other words, utility is not limited to the question of how much races help us to acquire knowledge and develop science. Instead, reference to race and racial groups can also be useful if it helps us to improve our social, political, and moral circumstances. Hence, both epistemic and non-epistemic utility must be equally considered when determining the reality of race and racial groups.

Notice that Spencer’s BRR rejects this philosophical move and the role of non-epistemic considerations in metaphysics. His “parity of reasoning” argument illustrates this point. Spencer compares race with paradigmatic examples of real kinds, such as TYRP1 gene and 93C allele. Another example is the kind *monophyletic group*. This kind is part of cladistics, a well-ordered scientific research program in biological classification. The kind monophyletic group is useful to define and classify groups of organisms according to the aim of cladistics, namely produce strictly genealogical classifications of organisms and species (Baum & Smith 2013; Wiley & Lieberman, 2011). This kind is real because biologists need it when developing generalizations, predictions, theories, explanations, and other activities in cladistics (2012, 190–191). According to Spencer’s parity reasoning, biological race realism is correct if races are analogous to monophyletic groups (2019a, p. 78). If the reality of the kind monophyletic group (and the individual monophyletic groups) in biology is justified solely based on epistemic utility, the same principle applies to the kind race (and the individual five races) in biology.

Using this line of argument, Spencer rejects social, political, and moral considerations in the metaphysics of real biological kinds. The problem is assuming that the reality of these kinds requires any non-epistemic justification (2012, p. 200). This requirement is too demanding for a theory of kinds *in science*. Spencer claims that paradigmatic examples of those kinds (e.g., genes, alleles, TYRP1 gene, 93C allele, monophyletic group) do not require non-epistemic utility. Scientists do not justify the reality of such kinds with statements about how socially, politically, or morally useful those entities are. Hence, there is no reason to ask them to justify the reality of race and racial groups in the same way.

The discussion above reveals a commitment underlying Spencer’s BRR. According to him, non-epistemic values *should not* influence the acceptance or rejection of race and racial groups as real biological kinds. In the next section, I challenge this conclusion. I argue that social, political, and moral judgments can and should play an indirect role in determining the reality of races in biology. My analysis culminates with broader questions about the relationship between metaphysics and values. These questions have been rarely considered by metaphysicians of race (c.f., Malon, 2006, 2022; Ludwig, 2016).

## Risk and non-epistemic values in biological race realism

The work of Heather Douglas and other recent philosophers of science offers tools for identifying how non-epistemic values have a place in BRR that has not been appreciated so far (Biddle, 2016; Biddle & Kukla, 2017). Douglas formulates a contemporary version of the *inductive risk argument,* an argument originally sketched by Rudner (1953). Inductive risk is the chance that scientists might be wrong in accepting or rejecting a hypothesis (Douglas, 2000, p. 561). As scientists’ conclusions result predominantly from inductive reasoning, there is an inevitable gap between scientific evidence and conclusions. Scientists cannot be entirely sure about their conclusions. For this reason, scientists will face the risk of error: they might reject a true hypothesis (false positive) or accept a false hypothesis (false negative).

This risk of error motivates scientists to assess the possible consequences of such errors. Some consequences might involve harm to the production of scientific knowledge, but in many cases the harm is to society as a whole. For example, imagine that scientists investigate whether chloroquine can cure patients of COVID-19. If chloroquine cures patients, but scientists conclude otherwise, these scientists will harm society. People would be discouraged from getting a fast, cheap, and easily accessible cure for the virus COVID-19. Now imagine if chloroquine is not effective against COVID-19, but scientists conclude that it is. This conclusion will also harm society. This time, people might buy chloroquine without realizing its serious side effects. For example, chloroquine can cause rhythmic heart problems and worsen diabetic conditions.

Scientists will assess these two types of consequences before concluding whether chloroquine is effective or not against COVID-19. They will consider how much harm wrong scientific conclusions might cause. Scientists want to minimize the risk of defending and spreading these wrong conclusions, especially when they can cause great harm. For example, if chloroquine represents a reasonable risk to human health, scientists will make sure to gather as much data as possible. They will conduct very rigorous experiments before concluding that chloroquine is effective against COVID-19.

By weighing the risk of error, scientists are implicitly letting social, political, and moral judgments influence their work. Scientists imply that certain actions are more harmful to society than others, for example. According to Douglas, these non-epistemic judgments have an *indirect* influence on how scientists arrive at their conclusions because these judgments might “act to weigh the importance of uncertainty about the claim, helping to decide what should count as sufficient evidence for the claim” (2009, p. 96). In other words, non-epistemic values influence how much evidence scientists need before concluding that chloroquine cures COVID-19. All else being equal, the more harmful a scientific conclusion is, the more evidence is needed before advocating this conclusion. Scientists must make sure that this conclusion is not wrong and thus avoid causing unnecessary harm to society.

These considerations about inductive risk suggest a way to challenge Spencer’s BRR. While one might agree with him that social, political, and moral judgments do not count as reasons for claims such as “X is a biological race,” one might still argue that those judgments *indirectly* influence such claims. Thus, the problem is that Spencer fails to recognize that non-epistemic judgments can have this influence and, in so doing, he fails to identify limitations of his parity of reasoning argument.

To expose the problems with Spencer’s view, let us return to the comparison between race and monophyletic group (Sect. 3). Monophyletic group is a paradigmatic example of real kinds, figuring in theories and generalizations of a well-established research program (cladistics). Scientific conclusions about monophyletic groups involve inductive risk because scientists typically rely on inductive evidence before concluding that “X is a monophyletic group.” If scientists are wrong about this claim, non-negligible consequences may result in those areas of study. For instance, one might have to revise large chunks of biological classification, which recently happened to dinosaur classification (Baron et al., 2017). Still, being wrong about “X is a monophyletic group” would likely have a minor impact on society broadly construed.

The case of race is much more complex. Let’s consider the claim that “X is a biological race.”^9^ If scientists are wrong about this claim, severe social consequences might unfold. For instance, consider a case in which scientists might use race as a proxy for studying disease susceptibility and eventually lead to drug development (Risch et al., 2002).^10^ If scientists are wrong about “X is a biological race,” medical applications of genetics will get worse and harm individuals’ access to health care (Bamshad et al., 2004). Population geneticists are aware of the risks when grouping humans explicitly in terms of race and human continental population (Bamshad et al., 2004; Bliss, 2012; Foster & Sharp, 2004; Lewis et al., 2022; Yudell et al., 2016). For instance, while scientists can legitimately divide humans in different ways depending on the research context, the emphasis on the five human continental populations might wrongly suggest that these populations are genetically more “fundamental” or “real” than alternative ones (Foster & Sharp, 2004, p. 795). This idea can lead to stereotyping and people might have their social identities challenged. These harmful consequences are even worse if they are preventable and come from mistakes made by scientists.

Given the inductive risk argument, scientists should include those worries in the inductive risk analysis. As geneticists discover patterns of reproduction among humans and extinct humanoids, they should worry about how these discoveries will become part of the racial discourse in the US, how they will be represented, and how they might feed into racism. For instance, minority groups such as Melanesians might be harmed by racial stereotypes that associate the biological patterns of reproduction (i.e., the presence of “ancient” DNA in those groups) and ideas of cultural primitivism, etc. (Havstad, 2022). These non-epistemic value considerations should inform how much evidence is gathered by scientists and how they conduct their work. In fact, there is extensive evidence that scientists take non-epistemic value considerations seriously (Committee on the Use of Race, E., National Academies of Sciences, Engineering, and Medicine, & Committee on Population, 2023).

Inductive risk is not the only type of risk discussed by philosophers of science (Elliott & Richards, 2017). *Epistemic risk* corresponds to unintended consequences that could result from possible mistakes in any epistemic activities in a research project (Biddle, 2016; Biddle & Kukla, 2017). Scientists can make mistakes at different research stages, such as when analyzing results or even gathering data. Recall that sampling bias was precisely one of the criticisms raised against the work of Rosenberg and colleagues (Serre & Paabo, 2004; Tischkoff et al. 2009). In this sense, inductive risk (mistakes in inferring a hypothesis) is only a sub-type of epistemic risk. In turn, *ethical risk* is a separate type of risk and it concerns harmful consequences that unfold from science regardless of potential mistakes (Biddle & Kukla, 2017, p. 219). For instance, clinical studies involve a perennial risk of harming or compromising the autonomy of their participants. These risks come from the very nature or design of those studies, and they cannot be dismissed even if such studies were epistemically flawless. Research programs on racial and gender differences frequently involve significant ethical risks because the methods and experiments in these programs contain racist and sexist assumptions (Brown, 2017). These studies might solidify racist and sexist practices in society.

The studies conducted by Rosenberg and colleagues are not free from ethical risk. For example, these studies use linguistic terms (“Black,” “Asian,” etc.) that are identical to the ones present in the OMB racial classification and the everyday racial discourse in the US (and elsewhere). For this reason, such studies may contribute to reinforcing beliefs about the essentiality and immutability of racial groups and human identity (Gannett, 2004; Wills, 2017). This type of ethical risk is most likely absent in research involving the monophyletic groups. Rosenberg and colleagues (2002) seem aware of the significant ethical risks involved in their research and explicitly try to distance themselves from racial discourse. To mitigate some of those risks, Rosenberg and colleagues try to reassure the public that their research is *not* about race (2005).^11^

More generally, one should not underplay the epistemic and ethical risks in population genetics research. The models, data, and results of population genetics influence the DNA Ancestry test industry, which feeds into popular conceptions of race. These tests can shape people’s group identities through an idea of shared genetic ancestry, leading them to revisit their identity and react to the genetic knowledge in various ways (Panofsky & Donovan, 2019; Reardon & Tallbear, 2012; Roth & Ivemark, 2018). In this process, the abstract genetic clusters from population genetics are interpreted as cultural and historical groups (Blom, 2022). This process reinforces the view of groups as biologically and culturally homogeneous, and culture itself is seen as biologically rather than environmentally determined (Tallbear, 2013). Thus, while scientists might try to reassure the public that their work is not about race, old and pernicious ideas about genetic determinism and essentialism might live on. These ideas can feed racism, extremism, and different forms of injustice (Haddad, 2023; Jackson, 2022; Kampourakis & Peterson, 2023).^12^

Let’s take stock. So far, I have shown that epistemic (including inductive) and ethical risks are present in the studies conducted by Rosenberg and colleagues Rosenberg et al. (2002). These studies carry significant risk, requiring those geneticists to be extra careful in their strategies to mitigate possible errors and recognize the ethical implications of their work. These risks and implications are even higher when we consider downstream applications, such as the use of population genetics models in the industry of DNA Ancestry tests. Other types of research (e.g., on monophyletic groups) involve much lower risks and implications. Spencer’s “parity of reasoning” argument (Sect. 3) simply does not consider how different kinds involve different levels and types of risk. His argument emphasizes that both kinds are real if they have modest epistemic utility, but it fails to notice how non-epistemic factors impact the use of each kind in science. Claims such as “X is a monophyletic group” and “X is a biological race” depend on non-epistemic value considerations. Many scientists know that, and this leads them to be more cautious about the latter claim than the former. This caution prompts scientists to carefully revise their work to avoid producing unnecessary harmful consequences to society (Douglas, 2003). In the remainder of this section, I argue that Spencer’s theory should demonstrate a similar sensitivity to non-epistemic value considerations.

To be fair, Spencer is aware of two ethical risks related to BRR (2018b, 2019a).^13^ First, white nationalists and other racist actors might attempt to use his theory for their political purposes. This appropriation is already underway, and it comes as no surprise (Jackson, 2022). After all, while population geneticists including Rosenberg et al., (2002, 2005) are reluctant to even use the term “race” in their research, Spencer defends the legitimacy of a biological understanding of race and racial groups. In fact, flawed linguistic assumptions suggest that geneticists are talking about race even if they do not recognize it (Sect. 2). Second, a possible consequence of biological race realism is reinforcing psychological essentialism and, therefore, racist beliefs. As empirical studies indicate, claims such as “X is a biological race” lead people to think that racial divisions are fundamental and necessary aspects of reality (Donovan, 2014, 2016, 2017; Ludwig, 2016). When students are confronted with those claims in the context of a biology classroom, they tend to develop essentialist views of races and racist beliefs. Old-fashioned ideas that some humans are essentially more intelligent or altruistic than others gain traction.

Spencer does not give much attention to the risk of appropriation, but he addresses the worry of BRR promoting racist beliefs (2019a). He takes a “scientifically informed approach” to this issue and argues there is still no strong evidence for the link between BRR and racism in the classroom (2019a, p. 240). As Spencer notices, the work of Donovan (2014, 2016, 2017) suggests that racist beliefs depend on the degree of a student’s comprehension of Mendelian genetics. Hence, he concludes, “perhaps one morally respectable way to do philosophy of race is not to suppress research on biological race realism, but rather, to improve the public’s understanding of genetics” (2019a, p. 240). This conclusion might seem plausible at first. As one of the reviewers pointed out, the idea that education can be an effective measure against racism is not new, but its efficacy needs further evidence (Brattain, 2007; Donovan et al., 2024). After all, racism involves complex and interrelated dimensions, such as structural but also ideological and interpersonal aspects (Bonilha-Silva, 1997; Collins, 2008). Spencer’s “scientifically informed approach” must consider how effective education is against these aspects. The lack of consideration here is another problem with his approach.

Spencer’s conclusion above is telling. It indicates that he is somewhat worried about the ethical implications of BRR. His way of dealing with those ethical implications resembles strategies that scientists take when dealing with ethical risks that “may function as sufficient reasons to block a research project altogether, or they may shape its methodology or implementation in myriad ways” (Biddle & Kukla, 2017, p. 219). Ethical risks might give scholars reasons to either pursue or abandon a research project.^14^ Currently, Spencer does not think there is enough evidence to abandon BRR as a research project, but I suggested that sociological literature might indicate otherwise (see also Jackson, 2022). At the very least, Spencer seems to acknowledge the role of ethical risks in his metaphysics. Two questions arise at this point. One is whether Spencer’s BRR involves other (unacknowledged) ethical risks. Another issue is whether this view acknowledges other types of risk, such as epistemic risk. I focus on the latter point below.

Spencer’s BRR involves epistemic risk in case Spencer can be mistaken and these mistakes can have foreseeable harmful consequences. This is indeed the case. For example, the results proposed by Rosenberg et al. (2002) rely on numerous decisions about study design, as well as what and how to represent genetic diversity. Scientists show awareness of this fact by discussing the limits of applicability of those representations (Rosenberg, 2011; Rosenberg et al., 2005). Misrepresentations can easily unfold if they are overemphasized or extended beyond those limits. However, Spencer’s metaphysical conclusions overly emphasize a particular result and representation (i.e., K = 5) beyond what scientists do. After all, Spencer ignores all the other alternative classifications (K = 2, K = 3, etc., but also classifications using other methodologies) and misrepresents K = 5 as a privileged level of genetic subdivision (Winsberg, 2022). Spencer must explain why this particular representation must be priviledged in arguments about the reality of race. This is not the only mistake in Spencer’s analysis. For instance, one might argue that the set of studies done by Rosenberg and colleagues is not representative of the work and goals of most population geneticists (Sect. 2). In this case, Spencer might be mistakenly treating Rosenberg’s work as paradigmatic of population genetics, resulting in a misinterpretation of population genetics and its epistemic aims. Finally, Spencer’s theory of biological kinds might be mistaken in the sense that it fails to meet what Spencer himself considers to be the goal of any adequate theory of kinds. The goal would be to capture most if not all cases, of what scientists take to be legitimate scientific categories (Spencer, 2012, 2019a). Indeed, Winsberg (2022) suggests that Spencer’s theory fails to be adequate. All these possible mistakes by Spencer constitute epistemic risks with the foreseeable harmful consequence of reinforcing beliefs in the existence of biological races in the public and weaponizing extremist groups (Jackson, 2022).

These considerations bring us to the question: how should epistemic risks legitimately influence metaphysical theorizing, such as Spencer’s theory of race? I contend that responsible metaphysics of race must at least leave open the possibility that epistemic risk influences metaphysical reasoning. The problem with Spencer’s BRR is that this possibility is not available. For instance, imagine that empirical studies show a clear and indisputable link between BRR and the promotion of racist beliefs in only a few circumscribed contexts. At the same time, imagine that there are some good reasons to keep researching BRR and that pilot educational practices are starting to be developed, such that there is a chance to mitigate those racist beliefs and their consequences. One might decide to keep pursuing BRR under these conditions, but this situation involves higher epistemic risk than if no link between BRR and racist beliefs exists. The existence of such a risk would morally demand adjustments in how the metaphysics of race is conducted. While epistemic risk might not motivate Spencer to change aspects of his theory of real biological kinds, it should motivate him to carefully re-examine the allegedly epistemic utility of race and racial groups. In the face of high epistemic risk, the reality of race and racial groups must depend on extremely clear and convincing cases of epistemic utility.^15^

This conclusion indicates that non-epistemic values can and should indirectly influence BRR. In the hypothetical case of a clear link between BRR and racist beliefs, the epistemic risk associated with BRR has harmful social and political consequences, namely the promotion of those beliefs. Hence, before concluding that “X is a biological race,” Spencer must explicitly assess how bad it would be to promote racist beliefs based on a mistake. This assessment will indicate to him how careful his analysis of epistemic utility must be.

Spencer’s analysis is not convincing, especially when we consider the epistemic and ethical risks involved in BRR. As discussed in previous sections, Spencer characterizes the epistemic utility of race and racial groups as their capacity to ground generalizations in population genetics. Nevertheless, Spencer fails to consider how other levels of grouping can better explain the same phenomena accounted for by race and racial groups (Kalewold, 2024). Moreover, Spencer’s actual example of epistemic utility is extremely modest in at least two ways. First, racial terms (terms for human continental populations) figure in hypotheses about the very reference of those terms (2019a). Most, if not all, terms in science are useful in this way, particularly in the context of exploratory research. Hence, it is unclear to what extent reference to race and racial groups is distinctively useful. Spencer does *not* provide further examples.^16^ Second, Spencer treats population genetics as a research program, but his focus is only on the set of studies conducted by Rosenberg and colleagues. One way to mitigate epistemic risks would be to broaden the scope, looking for other studies that might indicate the epistemic utility of race and racial groups. However, Spencer does not offer us this analysis. It is an open debate whether the reference to race and racial groups matters outside the specific research agenda of Rosenberg and colleagues. Furthermore, that research agenda has been heavily criticized in several ways, but Spencer does not consider how these criticisms can raise doubts about epistemic utility (Sect. 2).

At this point, one might argue that race and racial groups matter in other domains, such as race-based medicine (Hardimon, 2017; Spencer, 2018a). To offer a detailed discussion of this area would drastically influence the scope of the paper. Instead, I limit my discussion to raising the problem of co-reference. Racial terms might be present in population genetics, medicine, and other areas of science. Reference to race and racial groups might help Rosenberg and colleagues to study genetic clusters, while also helping clinicians offer fast diagnoses to their patients. One might be tempted to say that the same biological kinds (race and each racial group) are useful in different ways and contexts, but this temptation might result in a mistake. Only if the kind (e.g., “Asian”) has the same reference across research contexts can one lump together the benefits of this kind. Co-reference is necessary if the uses of that same term are supposed to capture the same “real” kind. The problem is that this co-reference is yet to be proven. Again, the conclusion is that, given the epistemic risk involved in his defense of BRR, one should carefully examine the epistemic utility of racial categories. This utility seems even more modest and less clear than Spencer himself admits.

This conclusion resembles one of the main criticisms against BRR (Sect. 3). According to this criticism, racial divisions are not sufficiently useful to be deemed “real.” Spencer replied to this idea by arguing that real biological kinds do not have to be significant or central to science. Spencer’s metaphysical theory is thin, accepting many kinds and entities as “real.” Nevertheless, particularly when high ethical and epistemic risks are involved, the reality of kinds must be grounded on a clear, comprehensive, and convincing assessment of their epistemic utility. Spencer fails on this front (Kalewold, 2024; Winsberg, 2022).

Notice how my argument differs from Kitcher’s views (Sect. 3). Kitcher claims that race and racial groups are legitimate if there are more beneficial than harmful societal consequences when claiming so. For him, epistemic and non-epistemic values are equally important as they offer justificatory reasons for the reality of biological races. BRR is true only after weighing all the epistemic and non-epistemic consequences. On the one hand, I agree that possible consequences of claims as “X is a biological race” influence the metaphysics of race. Assessing these consequences enables one to understand the seriousness of the epistemic risks involved in that metaphysics. On the other hand, the influence of epistemic and ethical risk in the reality of races is indirect. Risks are not justificatory reasons for or against BRR, but they motivate us to re-examine and revise those reasons.^17^

Spencer fails to recognize this indirect influence of non-epistemic values in the metaphysics of race. He fails to recognize that the risks of BRR can and should legitimately influence metaphysical claims. These risks are high, while the minimalist version of BRR is explicitly a deflationary view with low explanatory power, philosophical gains, and numerous problems as discussed in previous sections (Kalewold, 2024, Bernstein forthcoming). There is simply little evidence of the epistemic utility or accuracy of BRR (and even most geneticists do not adhere to it). In situations like this, non-epistemic value considerations become particularly important. They can guide metaphysicians into understanding the potential *consequences* of their views. Spencer’s realism is not epistemically significant, but its consequences are potentially harmful. These considerations from epistemic and ethical risk present here should be enough to put Spencer's theory finally to rest.

More generally, I contend that our metaphysical theories should be open to the influence of non-epistemic values. The work of scientists and other professionals is open to such influence, helping them to take precautions, safeguards, and responsible attitudes toward complex issues. Non-epistemic values guide these actors, helping them to ensure social responsibility (Douglas, 2003, 2009). The same should apply to the work of metaphysicians. Or, alternatively, metaphysicians of race must at the very least explain why their work should *not* be subject to such an influence. This explanation would set them apart from scientists and many other professional groups that deal with risks all the time (Douglas, 2009). It would also raise a broad question concerning social responsibility. What does it mean for the metaphysics of race to be done in a socially responsible way? What types of influence from non-epistemic values can ensure this responsibility? To what extent non-epistemic utility matter and should guide socially responsible work in the metaphysics of race? These are fundamental questions once we recognize that metaphysics of race and other branches of metaphysics do not operate in a silo.

## Conclusion

In this paper, I examined Spencer’s BRR and argue that it fails to sufficiently consider how social, political, and moral values influence the metaphysics of race. Spencer is not alone in this point. The relationship between metaphysics and non-epistemic values is underexplored by philosophers and deserves careful analysis. If one agrees that the metaphysics of race involves epistemic and ethical risks, one should consider how these risks legitimately influence the decisions, reasoning, and conclusions of metaphysicians.

An exhaustive analysis of this influence is beyond the scope of this paper. Instead, my goal here was threefold. First, I surveyed numerous problems faced by Spencer’s BRR. These problems are enough to reject Spencer’s theory, but they do not go as far as to discuss the relationship between BRR and non-epistemic values. A key contribution of this paper is to explore this relationship. Second, I showed that risks can and should legitimately influence the metaphysics of race, at least in the sense of demanding high levels of scrutiny, clarity, and evidence for the alleged epistemic utility of race and racial groups in science. Spencer’s theory fails this level of scrutiny. This failure is a final nail in the theory’s coffin. Third, my analysis of BRR should be an invitation for metaphysicians to consider the broad question of socially responsible metaphysics. After all, should metaphysics be sensitive to social and political risks? If so, are these risks only relevant for ameliorative metaphysical projects, or do they also apply to descriptive projects? But how?

## Footnotes

1 Framing Cystic Fibrosis as a “White disease” is a problem for several reasons. The use of racial language has historically contributed to the underdiagnosis of Cystic Fibrosis in non-white populations (Yearby, 2021). Thus, the epistemic utility of racial classifications in this case is disputable. I delve into the weaknesses of minimalist later in the paper.

2 I will refine this statement later in the paper. For now, it is worth noticing that authors adopt different terminology when referring to biological kinds. Recently, Spencer adopted “genuine biological entity” (2019a). I ignore these mere terminological variations and retain the term “real biological kinds.” What matters is the theory of kinds proposed by the authors rather than the label used.

3 One might dismiss this argument by saying that it incorrectly assumes a distinction between epistemic and non-epistemic values (Longino, 1996). While I acknowledge that distinguishing types of values is problematic in many ways, at least sometimes these distinctions are still philosophically useful because they help us to understand approximately how values influence science. Evidence of this philosophical usefulness is the fact that most debates about values in science have not completely abandoned those distinctions (e.g., Brown, 2013; Douglas, 2009; Intemann, 2005; Steel, 2010).

4 Ludwig (2016) has briefly explored some of these influences, but it does not engage with the risk literature as I do here (see below). Moreover, in a different (non-metaphysical) context, the literature on pragmatic encroachment and ethics of belief has been advancing similar arguments to the ones presented in this paper. For instance, Rima Basu (2023) raises the question of how philosophical work in general should be mindful of moral and sociopolitical risks. It is worth noting that my defense of the interconnection between metaphysics and ethics has a long tradition in continental feminism and the philosophy of science (e.g., Trappes, 2019).

5 The semantic assumption enables Spencer to privilege a specific level of analysis in genetic studies. While these studies create genetic clusters at different levels of resolution, those studies do not associate any level of clustering with racial categories. As it will become clear later, Spencer makes the association between these categories and a particular level of clustering. What grounds this association is the semantic assumption above.

6 However, this prediction is more or less accurate depending on how self-declaration works. For example, if someone self-declared as Asian, predictions might systematically fail because that person might fall into the Eurasian category (as it includes South and Central Asian). Prediction is more accurate if self-declaration forms are framed in terms of subcontinental categories, such that individuals have to declare as South, Central, or East Asia.

7 For example, population geneticists argue that grouping humans in terms of finer-grain ancestry groups or even using similarity rather than ancestry is a much more informative approach (Lewis et al., 2022).

8 Catherine Bliss (2012) shows convincingly that scientists are sophisticated and strategic when talking about race. Scientists such as Noah Rosenberg, Marcus Feldman, and David Reich consider genetics an important tool for social justice, addressing health disparities between socially constructed races. Thus, scientists explore the relationship between genetics and race, but the notion of race here is a social constructionist one.

9 For the sake of the argument, let’s assume that “X is a biological race” is synonymous to “X is one of the five human continental populations.” This assumption helps us to understand that, even if we accept Spencer’s flawed linguistic assumptions, his argument faces many problems.

10 The use of race in biomedicine is a highly controversial debate, as several scholars argue about the potential benefits and harms of that use. For recent treatment on the issue, see Malinowska and Serpico (2023).

11 Some population geneticists emphasize the distinction between race and ancestry, while others tend to be ambiguous about it (Reich 2018a, 2018b). One example of the latter case is David Reich (Harvard). He is also among the scientists who emphasize the epistemic gains associated with referring to human continental populations.

12 This is a far-limited account of epistemic and ethical risks in population genetics and its downstream consequences. A much more comprehensive account exceeds the scope of this paper and it would have to consider, for instance, how epistemic biases in the field might come from the fact that population genetics seems disproportionately white and male.

13 The two issues are mentioned both in print and interviews, as the interview below: https://biopoliticalphilosophy.com/2020/05/20/dialogues-on-disability-shelley-tremain-interviews-quayshawn-spencer-redux/.

14 Suppressing research is also an ethical risk that must be avoided. Hence, it is important to weigh ethical risks for and against a research program.

15 This clarity depends on considering to what extent race and racial divisions have been useful in ideological and historical rather than epistemic ways. The worry here is that race might persist in science due to non-epistemic and malefic functions rather than its allegedly “epistemic usefulness.”

16 Furthermore, as described by Winsberg (2022), Spencer’s examples show that the dividing humans into continental populations is an useful activity, but it does not show that any particular group (e.g., Asian) is itself useful. Hence, the reality of populations described by particular categories is unclear.

17 It is worth noticing that some philosophers dispute the distinction between the direct and indirect influence of epistemic risk (Elliott 2011). If these philosophers are correct, my line of argument might not be so different from Kitcher after all. Nevertheless, this would only reinforce that Spencer (2012) is too dismissive about the intricate relationship between metaphysics and values when he replies to KitcherKitcher.
